# Controversies in regenerative medicine: Should intervertebral disc degeneration be treated with mesenchymal stem cells?

**DOI:** 10.1002/jsp2.1043

**Published:** 2019-03-01

**Authors:** Markus Loibl, Karin Wuertz‐Kozak, Gianluca Vadala, Siegmund Lang, Jeremy Fairbank, Jill P. Urban

**Affiliations:** ^1^ Department of Spine Surgery Schulthess Klinik Zürich Switzerland; ^2^ Department of Trauma Surgery Regensburg University Medical Center Regensburg Germany; ^3^ Institute for Biomechanics, Department of Health Sciences and Technology ETH Zürich, Zürich Switzerland; ^4^ Spine Center, Schön Klinik München Munich Germany; ^5^ Academic Teaching Hospital and Spine Research Institute Paracelsus Private Medical University Salzburg Austria; ^6^ Department of Health Science University of Potsdam Potsdam Germany; ^7^ Department of Orthopaedic and Trauma Surgery Campus Bio‐Medico University of Rome Rome Italy; ^8^ Nuffield Department of Orthopaedics Rheumatology and Musculoskeletal Sciences (NDORMS), University of Oxford Oxford UK; ^9^ Department of Physiology, Anatomy and Genetics University of Oxford Oxford UK

**Keywords:** degenerate disc disease, inflammation, intervertebral disc degeneration, mesenchymal stem cells, microenvironment, regeneration

## Abstract

Low back pain (LBP) can significantly reduce the quality of life of patients, and has a considerable economic and social impact worldwide. It is commonly associated with disc degeneration, even though many people with degenerate discs are asymptomatic. Degenerate disc disease (DDD), is thus a common term for intervertebral disc (IVD) degeneration associated with LBP. Degeneration is thought to lead to LBP because of nerve ingrowth into the degenerate disc, inflammation, or because degradation of extracellular matrix (ECM) alters spinal biomechanics inappropriately. Thus, while the objectives of some interventions for LBP are to control pain intensity, other interventions aim to deal with the consequences of disc degeneration through stabilizing the disc surgically, by inserting artificial discs or by repairing the disc biologically and preventing progressive IVD degeneration. Despite tremendous research efforts, treatment of LBP through the use of regenerative interventions aiming to repair the IVD is still controversial. The use of mesenchymal stem cells for IVD regeneration in a patient‐based case will be discussed by an ensemble of clinicians and researchers.

## 1 BACKGROUND

Low back pain (LBP) can significantly reduce the quality of life of patients,^1^ and has a considerable economic and social impact worldwide.^2^ It is commonly associated with disc degeneration, even though many people with degenerate discs are asymptomatic.^3^ Degenerate disc disease (DDD), is thus a common term for intervertebral disc (IVD) degeneration associated with LBP. Degeneration is thought to lead to LBP because of nerve ingrowth into the degenerate disc, inflammation, or because degradation of extracellular matrix (ECM) alters spinal biomechanics inappropriately. While the objectives of some interventions for LBP are to control pain intensity, other interventions aim to deal with the consequences of disc degeneration through stabilizing the disc surgically, by inserting artificial discs or by repairing the disc biologically and preventing progressive IVD degeneration. Despite tremendous research efforts, treatment of LBP through the use of regenerative interventions aiming to repair the IVD is still controversial, as here discussed by clinicians and researchers. Taking account of the diverse results of preclinical and clinical trials as well as the different experiences of the authors on LBP treatment, this article is structured as “Yes” and “No” chapters, arguing in favor or against the use of mesenchymal stem cells (MSCs), respectively. In order to give a high priority to the translation of research to clinical application, this article will focus on the evaluation of MSCs. Noteworthy, other cell types like induced pluripotent stem cells (iPSC) are on the rise in preclinical trials but will not be subject of this manuscript.

## 2 THE CASE

**Figure 1:**
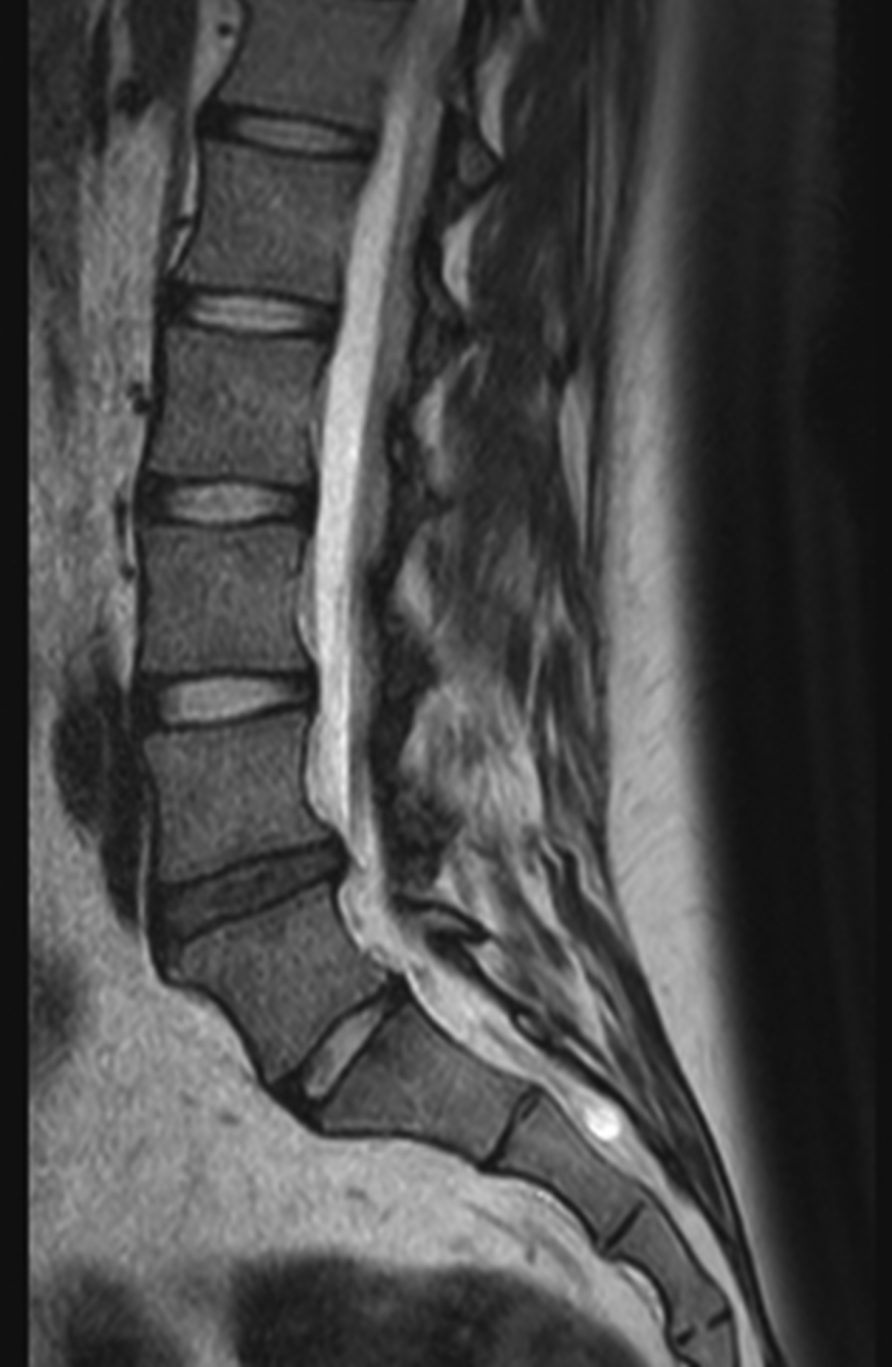
T2‐weighted magnetic resonance imaging (MRI) sagittal reconstruction of a 22‐year‐old female with persistent LBP for 8 months despite conservative treatment and IVD degeneration at the L4/5 level Pfirrmann grade III without endplate changes

This is a T2‐weighted magnetic resonance imaging (MRI) sagittal reconstruction (Figure 1) of a 22‐year‐old female with persistent LBP for 8 months despite conservative treatment (physical and medical). The MRI reveals IVD degeneration at the L4/5 level Pfirrmann grade III without endplate changes (MODIC). The annulus fibrosus is intact with preserved disc height of at least 75%. Segmental instability and isthmus pathology were excluded. The patient is normal weight with a BMI of 28 kg/m^2^.

## 3 YES

### 3.1 The importance of selecting a suitable MSC subpopulation for IVD regeneration

MSCs can be isolated from bone marrow or adipose tissue and are recognized as a feasible donor cell candidate for regenerative therapies because of their accessibility and proliferation characteristics.^4^, ^5^ The classical approach to identify MSCs is via a set of surface markers, for example, CD90+, CD73+, CD105+, CD14−, CD34−, CD45−, CD79−, or CD19 and HLA‐DR‐, according to the International Society for Cellular Therapy (in addition to plastic adherence and differentiation capability).^6^ However, this approach yields a relatively heterogeneous population with progenitor cell characteristics. It recently became increasingly clear that preselection of particularly suitable MSC populations can further enhance the clinical outcome. CD271‐ MSCs, for example, have a higher potential for nucleus pulposus (NP)‐like differentiation than their CD271+ counterparts and may hence be primarily used in the future.^7^ In cartilage research, a shift towards subpopulations has already taken place. Perez‐Silos et al summarize the recent findings and highlight that CD73^+^ and CD73^+^CD39^+^ cell subpopulation evidenced high chondrogenic potency.^8^ These results may also be of relevance for IVD research. Overall, some of these developed techniques and approaches may also substantially help to promote clinical success for DDD treatment.

### 3.2 Successful techniques to boost MSC survival in the harsh IVD microenvironment

There is no denial: The IVD represents a very harsh microenvironment even in its healthy state, due to low oxygen levels, high osmolarity, nutritional deficits and high mechanical loading. These conditions are further aggravated by acidity and inflammation during degeneration.^9^, ^10^, ^11^ In fact, the detrimental effects of this harsh IVD microenvironment on MSC survival and functionality were highlighted a decade ago^12^, ^13^ and confirmed by later studies.^14^, ^15^ However, not all microenvironmental challenges are detrimental for MSCs. In the contrary, moderate IVD‐like glucose and oxygen conditions seem to promote MSC differentiation, as evidenced by higher expression of relevant ECM proteins.^13^, ^15^ Based on these results, preconditioning/differentiation of MSCs could provide a viable solution and might improve the clinical success for patients.

Aside from the beneficial effects of exposing MSCs to certain IVD microenvironmental condition (eg, induction of hypoxia via cobalt chloride, a chemical inducer of HIF‐1^16^), preconditioning and differentiation of MSCs can be induced by a variety of other approaches: (a) Treatment of MSCs with specific induction medium is the most classical and well‐established approach. Recent research highlighted that the use of a certain growth factor combination (eg, TGF‐ β1 plus GDF‐5) is distinctively effective in promoting an NP phenotype.^17^ (b) Alternative methods include adenoviral/lentiviral gene delivery, for example, modulation of Wnt11 to promote expression of SOX‐9, aggrecan, and collagen type‐2.^18^

In the coming year, the major break‐through in MSC‐based regeneration of the IVD may arise from a technique that has been hailed as the discovery of the century: CRISPR/Cas9 genome editing. CRISPR/Cas9 could for example be used to protect MSCs from the inflammatory IVD microenvironment by repressing the expression of cytokine receptors.^19^, ^20^ Other possible applications of MSC‐targeted genome editing with excellent potential for IVD regeneration may entail inhibition of cell senescence (eg, by inhibition of p16 by CRISPRi) as well as activation of extracellular matrix protein expression (eg, induced synthesis of aggrecan by CRISPRa).^21^

The development of novel techniques, ranging from sophisticated differentiation methods to genome editing, can lead to a massive improvement in MSC functionality and survival within the harsh microenvironment of the IVD, hence obliterating many of the concerns provided by the MSC opponents.

### 3.3 Pain reduction vs tissue regeneration: How much regeneration is really needed?

The therapeutic effect of transplanted MSCs for IVD degeneration is, at least in theory, multifold.^22^, ^23^ It can occur via differentiation of MSCs to NP cells, via the promotion of de novo extracellular matrix (ECM) production in the IVD and/or via reduction of IVD inflammation through the inherent anti‐inflammatory properties of MCSs.

Due to the nutritional deficits within the IVD, MSC‐based production of ECM proteins (or the stimulation of ECM synthesis by resident cells) is likely restricted and may not be sufficient to induce full IVD regeneration (see extensive elaborations under “No”). However, the goal of any patient will not be to have fully regenerated IVDs, but to be pain free. As there is increasing evidence for the relevance of inflammation in the development of discogenic back pain,^24^, ^25^ the anti‐inflammatory nature of MSCs may ultimately be more relevant than their regenerative nature. Reduction of inflammation may arise directly through anti‐inflammatory factors secreted by MSCs, but may importantly also occur via immunomodulatory paracrine effect of MSCs, affecting IVD cell cytokine expression as recently demonstrated by several research groups.^26^, ^27^, ^28^ Therefore, the fundamental question to ask: should not we stop focusing primarily on regeneration and emphasize pain reduction in preclinical and clinical studies?^29^

### 3.4 Preclinical studies: A valuable indicator for the therapeutic potential of MSCs

Everyone in the field knows: Animal models in IVD research are not ideal! Animals have smaller IVDs than humans and consequently a less harsh microenvironment, possibly with a different cell type (depending on the species). Nevertheless, it would be unwise to rigorously reject data gained in preclinical studies. In fact, the obtained results are valuable indicators of the therapeutic potential of MSCs—if evaluated with sufficient caution and a good understanding of the advantages and limitations of the various animal models. Especially studies in large animal models, ideally with naturally occurring IVD degeneration, are highly relevant for the field. While larger studies fulfilling these requirements are currently under way in various laboratories and results are eagerly awaited,^30^, ^31^ it was previously already shown that MSCs are able to promote disc regeneration in dogs with experimentally induced IVD degeneration.^32^ Importantly, similar results were observed in sheep, not only after nucleotomy,^33^ but also after annular injury^34^, ^35^ and annular incision,^36^ highlighting the therapeutic potential of MSCs in large animal models and hence possibly also in humans.

### 3.5 Which patients have been treated so far?

Six cohort studies investigated the feasibility and outcome after MSC transplantation in patients with DDD and chronic LBP (Table 1).^37^, ^38^, ^39^, ^40^, ^41^, ^42^ The mean age range in the different studies was 35 to 52 years. On radiological examination all patients revealed focal IVD degeneration with involvement of one or two segments of the lumbar spine. The Pfirrmann grading is reported for most of the studies and ranged from grade II to IV. After conservative management (physical and medical) of 3 to 6 months had failed to improve symptoms, patients were considered for MSC transplantation. Baseline pain and disability indexes revealed considerable lumbar pain with resulting disability in all patients. Only a limited number of patients also reported leg pain. Discography was performed in most patients to ascertain the symptomatic disc.

**Table 1:** Published clinical studies that suggest MSC‐injections for treatment of LBP attributed to DDD

	Number of patients	Inclusion criteria—Radiology	Cell source		Cell expansion time	Passage	Number of cells	Injected volume	Outcome Parameter	Mean age [years]	Treatment responder	Follow‐up time [months]	Additional substances
Kumar et al^37^	10	Discography, Pfirrmann III‐IV	Adipose‐derived mesenchymal stem cells	Autologous	21 days	3	25 × 10^6^ (n = 5) or 40 × 10^6^ (n = 5)	2 mL	VAS, ODI, SF‐36	43.5	6	12	Hyaluronic acid
Comella et al 38	15	Loss of height, Modic III	Adipose‐derived mesenchymal stem cells (SVF)	Autologous	No expansion	—	30‐60 × 10^6^	1 mL	VAS, ODI, Dalles pain questionnaire, functional, SF‐12, BDI, PPI	51.5	10	6	PRP
Noriega et al^39^	12	Pfirrmann II to IV	Bone marrow	Allogenic	27 ± 2 days	n/a	25 × 10^6^	2 mL	VAS, ODI, SF‐12	38.0	5	12	—
Elabd et al^40^	5	Protruding discs, discography, Modic endplate changes	Bone marrow	Autologous	Expansion time n/a	n/a	15.1‐51.6 × 10^6^	0.25‐1 mL	Quality of life questionnaire	40.4	2	48‐72	Autologous platelet lysate
Pettine et al^41^	26	Pfirmann IV‐VII, modic endplate changes, disc height	Bone marrow concentrate	Autologous	No expansion	—	2713 CFU‐F/mL	6 mL (2‐3mL per disc)	VAS, ODI	40 (Median)	21	24	—
Orozco et al 42	10	MRI (T2)disc height, water content	Bone marrow	Autologous	24 ± 4 days	3	10 ± 5 × 10^6^	n/a	VAS, ODI	35.0	7	12	

### 3.6 Do patients benefit from MSC transplantation?

A total of 78 MSC‐treated patients were included into the six cohort studies with a follow‐up time of 6 to 72 months. These patients displayed a mean significant improvement in Oswestry disability index (ODI) and visual analog scale (VAS) vs the controls (if present). However, this improvement seemed restricted to a group of 51 responders (65.4%).

In most studies, IVD height assessed by MRI did not recover, however, Pettine et al (2015) demonstrated an improvement of at least one Pfirrmann grade in eight patients at 12 months MRI follow‐up.^41^

### 3.7 Does origin and number of transplanted MSC matter?

Expanded MSCs from bone marrow^40^, ^42^ or adipose tissue,^37^ concentrated bone marrow cells^41^ and stromal vascular fraction (SVF) from adipose tissue^38^ were used as cell source for therapy approaches for disc degeneration. Bone marrow cells were reported to be expanded for 3 to 4 weeks in two of the six cohort studies. MSCs from adipose tissue were applied as expanded cells (for 3 weeks) and as SVF.

For logistic and financial reasons, Noriega et al (2017) investigated allogenic bone marrow derived MSCs (without immune matching) in a randomized controlled pilot trial.^39^ Improvements in pain (VAS) and disability (ODI) were observed starting at 3 months follow‐up, similar to the results with autologous cells.^42^

Cell number ranged from 10 to 60 x 10^6^ transplanted cells per disc. Pettine et al (2015) treated 26 patients with LBP with autologous bone marrow concentrate (BMC) injections with a follow‐up for at least 2 years.^41^ A significantly faster and greater reduction in ODI and VAS was reported in patients, who received greater than 2000 CFU‐F/mL. Therefore, these data indicate that the effectiveness of the transplantation is dependent on implanted cell concentration. On the other hand, Kumar et al (2017) found no statistical difference between a low‐dose and the high‐dose of autologous MSCs from subcutaneous abdominal adipose tissue with a final number of 20 × 10^6^ or 40 × 10^6^ cells/disc.^37^

### 3.8 MSC Transplantation is a safe procedure

Severe adverse events (SAEs) have not been reported in any of the six cohort studies.^38^ Elabd et al (2016) conducted a single center follow‐up study and examined five patients, previously treated with a single autologous intradiscal bone‐marrow derived MSCs injection after expansion under hypoxic conditions with 5% oxygen level.^40^ Neoplasms or abnormalities surrounding the treated region could not be found in the 4 to 6 years MRI follow‐up.

### 3.9 Summary “YES”

Major progress has been made in identifying suitable MSC sources, subpopulations and culture protocols, likely promoting the clinical success of MSC‐based IVD regeneration and thus improving today's outcome for the presented case compared to earlier times. Several preclinical studies demonstrated that MSC injection therapy is an effective treatment in promotion of disc regeneration and pain modulation.

The analysis of the literature does not allow a strong recommendation about MSC injection therapy yet. The few clinical studies showed MSC injection is a safe treatment option in patients with DDD presenting with persistent LBP for 6 months despite conservative treatment given intact annulus fibrosus and can be considered for the patient of the presented case. Analyzing all patients who received treatment in published clinical studies, not all profited equally from the therapy, indicating that patient selection according to yet unknown factors will be crucial.

## 4 NO

The idea of using MSCs to treat back pain appears very attractive. The treatment is simple, and minimally invasive, comprising of a single injection of a cell suspension into the disc. Tests on animals have been very encouraging with noticeable repair of degenerate nucleus pulposus within months after injection. Clinical tests too appear to relieve pain, and in some cases, show improvement in Pfirrmann grading of disc degeneration.

The underlying hypothesis for the use of MSCs, is that degeneration of the intervertebral disc is responsible for low back pain. The mechanisms by which MSC injections might relieve back pain, depend on assumptions of how disc degeneration leads to pain, and on the assumed effects of MSCs on the disc. Intervertebral discs may be intrinsically painful due to inflammatory processes and changes in pH leading to degenerative changes, which, in animal studies, appear to be modified by MSCs which are known to have immunomodulatory and anti‐inflammatory effects. The pathological changes may also affect the endplates, and MSCs are reported to modify the inflammatory milieu, changes in pH and reduced blood flow in this area as well. Finally, pain might be caused by the mechanical disturbance of a motion segment, such as disturbance of proprioception or facet joint degeneration. Intradiscal MSC injections, are thought to modify this origin of pain through enabling repair and regeneration of the disc's tissue and hence restoring the biomechanical behavior of the disc and surrounding spinal structures.^22^

However, there are several reasons for rejecting the use of MSCs for treatment for this illustrated case and for back pain in general.

### 4.1 Will the patient benefit from MSC injection?

**Figure 2:**
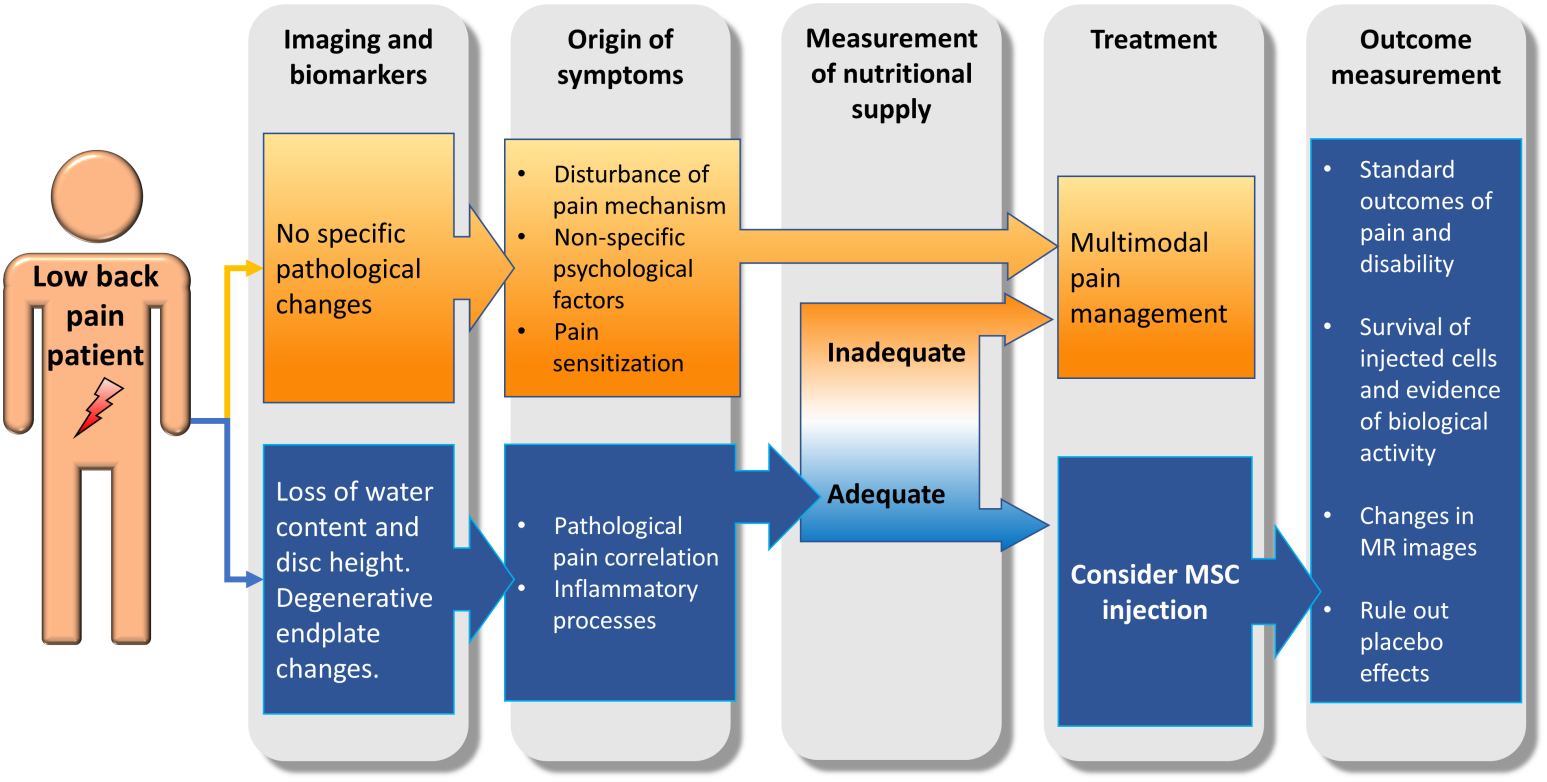
Clinical pathway to support the indication for MSC injection. Low back pain must be evaluated and correlated by imaging and supporting clinical tests. The treating physicians must rule out non‐specific pain sources and ensure a sufficient nutritional supply before considering MSC injection. Each treatment should be followed by outcome measurements that are attributable to the intervention

As shown in Figure 2, with MRI imaging and use of supportive clinical tests as the only means of diagnosis, choosing the right patient is difficult but essential. As MSC therapy would only help those patients whose pain arises from disc degeneration, the origins of the symptoms like disturbance in pain mechanism, pain sensitization or non‐specific psychological factors must be ruled out. Moreover, while there is some association between MRI gradings of disc degeneration and low back pain, many people with degenerate discs are asymptomatic.^3^ There is no consensus about the best diagnostic method and classification system to identify ideal candidates for regenerative IVD treatment.^22^, ^29^, ^43^ Hence whether the current case (Figure 1) might potentially benefit, cannot readily be determined.

### 4.2 Is there clinical evidence of the efficacy of MSC therapies?

For routine use of MSC therapies, the outcome must be attributable to the intervention, rather than non‐specific placebo effects, which are known to also be effective.^44^ The placebo effect is very strong for the treatment by means of injection into the disc.^45^ Hence, the evidence from randomized controlled trials (RCTs) using appropriate controls is essential before such treatments are routinely adopted. Eight clinical trials using MSCs to treat low back pain have been set up.^46^ None have reported any results so far.

Of the relatively sparse number of clinical studies using MSCs injection into the IVD to treat DDD, only one study finds that precursor cells, like MSCs are of no benefit in the treatment of IVD,^47^ whereas several other studies suggest MSC injection is a rational treatment option.^37^, ^38^, ^39^, ^40^, ^41^, ^42^ However, none of these studies have appropriate controls. Therefore, the placebo effect cannot be ruled out. In general, there are no multicenter studies and studies have only been on small numbers of patients. Also, the study protocols widely differ in the choice of inclusion criteria, the chosen cell sources for MSCs, the methods of transplantation and in the follow‐up conditions as reviewed by Sakai et al.^22^ Moreover, studies are missing specific outcome parameters, such as survival rate of injected MSCs and evidence of their biological activity. Potential harmful effects of MSC injections have also to be considered, though none have so far been reported. Cell leakage may induce osteophyte formation, as shown by Vadalà et al in an intervertebral disc degeneration rabbit model.^5^

### 4.3 Can MSCs remain viable post implantation?

For MSCs to be able to effectively perform their proposed roles of stimulating matrix production and inhibiting inflammation, they have to remain alive in the environment found in degenerated discs. They thus require a sufficient supply of nutrients, particularly glucose as, like native disc cells, MSCs are primarily glycolytic and die rapidly if glucose (but not oxygen) is removed.^48^ Efficient removal of lactic acid, the main metabolic product of glycolysis, is also essential as the acidic pH found in degenerated discs is detrimental to cellular activity and survival of both MSCs and disc cells.^12^

The disc is large and avascular and nutrients, necessary for survival of cells in the disc, are supplied by blood vessels at its margins. Concentrations of nutrients throughout the disc depend on the balance between supply of nutrients and cellular demand, so nutrient concentrations across the nutrient fall with distance from the blood supply. Hence, discs can only support a limited number of cells before nutrient concentrations in the disc center become too low to maintain cell viability. The cell density supported thus varies inversely with disc height^46^ with the cell density in rabbit discs for instance, being 30 to 40 times greater than that in adult human lumbar discs (cell density around 2‐3 × 10^6^ cells/mL in a normal human disc).

**Figure 3:**
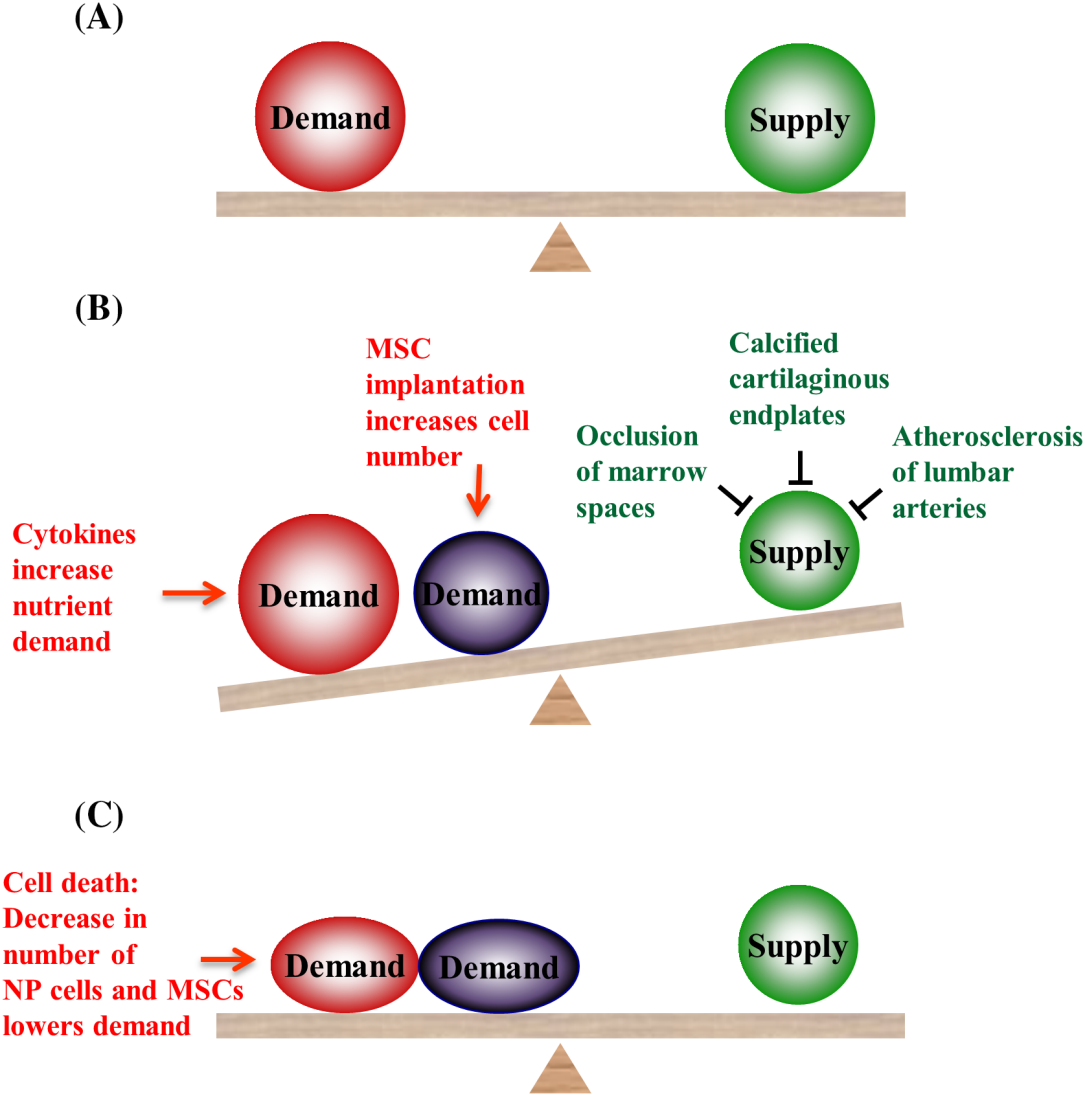
Influence of nutrient demand and supply on activity of MSCs and NP cells (a) Normal disc: rates of cellular demand and nutrient supply are in balance. (b) Degenerating discs: demand exceeds supply. The nutrient demand rises because cytokines stimulate cellular energy metabolism. An increase in cell number through MSC implantation would further increase the demand. The nutrient supply is diminished through such changes as calcification of the endplate, occlusion of marrow spaces and atherosclerosis of vertebral arteries. (c) Diminishing nutrient supply results in demand falling through decrease in cellular activity and/or cell death ‐ whether NP cells or MSCs would better survive is not known. (adapted from Huang et al^50^)

Nutrient supply is compromised in degenerated discs^49^ and hence cell density is lower than in normal discs as cells die until the drop in cell number balances the available and reduced supply (Figure 3).^50^ Implantation of MSCs would further disturb the nutritional balance since increasing cell number increases demand and further cell death would ensue with implanted MSCs competing with the remaining viable resident disc cells for available nutrients. Whether disc cells or implanted MSCs would better survive is unknown and currently there are no non‐invasive means of measuring cell density and cell phenotype in vivo.

Thus, if the nutrient supply is compromised and cannot adequately support additional cells, the implanted cells MSCs may die. Hence some means of determining the nutrient supply in potential patients, perhaps based on that suggested by Rajasekaran,^49^ should be developed.

### 4.4 Can surviving MSCs in the disc function adequately?

The constituent glycosaminoglycans (GAGs) of aggrecan are responsible for maintaining disc hydration and disc height.^51^ GAG loss is one of the first signs of disc degeneration. Hence restoring GAG concentration is a requirement for disc repair. The reported amount of GAG produced by MSCs varies widely with species, cell source and culture conditions; in recent studies of MSCs in pellet or gel culture GAG production ranges from 0.017 to 0.086 mg GAG/million cells/month.^7^, ^52^, ^53^, ^54^ GAG production rates increased more than 3‐fold by habituating MSCs to the harsh cellular microenvironment found in degenerate discs and through molecular biology techniques.^7^ Nevertheless, as the concentration of GAGs in the normal disc nucleus is around 70 mg/mL and since even the normal human lumbar disc cannot support more than around 3 million cells/mL,^46^ calculations show that it would take decades to restore the 25% of disc tissue (and hence of aggrecan) lost by the patient (Figure 1) through implanting even stimulated MSCs. The very slow repair of discs by MSCs might be explained by the findings that the half‐life of aggrecan in human discs is 4 to 6 years,^51^ and MSCs produce GAGs at only around 20 to 30% of the rate of normal NP cells.^54^

### 4.5 Alternately, could surviving MSCs stimulate resident disc cells to repair the disc or reduce production of inflammatory molecules?

Degenerating discs have a low cell density. Moreover, disc cells tend to produce GAG slowly because of the unfavorable environment and because many NP cells become senescent. Factors produced by implanted MSCs, if the cells survive at a sufficiently high density, could dampen down inflammation and improve the microenvironment and reduce pain. They may also produce factors which stimulate GAG production by the small remaining population of NP cells. However, this will come at a cost: stimulated cells demand more nutrients, again upsetting the balance between nutrient supply and demand and risking increased cell death. Currently it seems unlikely that implanted cells will survive at any significant density in degenerating discs which have already lost some of their resident cell population because of a compromised nutrient supply.

### 4.6 Why do tests in animals look so promising?

Animal experiments are usually carried out on young (even immature) animals. Their discs are much smaller than human discs and hence have a much higher cell density.^46^ Moreover, animal models of degeneration are usually acute, so the microenvironment is less hostile, with no reports of impairment of the nutrient pathway. In some species, the presence of notochordal cells (NC) in the NP is known to protect the IVD from the development of DDD, preserving structural integrity and biomechanical properties.^55^, ^56^, ^57^ A study by Matta et al pointed towards soluble proteins within notochordal cell‐derived conditioned medium that have the ability to maintain a healthy, proteoglycan rich NP and delay DDD in a canine model.^58^ For this reason, implanted cells are more likely to survive and remain active. Approaches to assimilate animal test settings to the complex degenerative situation in human DDD could involve the inclusion of inflammatory factors or the inhibition of vertebral endplate perfusion.^59^

### 4.7 Summary “NO”

Treatment of LBP by implantation of MSCs is unlikely to effect a significant repair or relieve pain. The poor nutrient supply to degenerate and even normal discs, severely limits the number of implanted cells which could survive. Hence MSCs are unlikely to reach concentrations able to inhibit inflammatory processes, whereas even limited matrix repair by MSC‐stimulation of surviving native disc cells or by MSCs themselves, is calculated to take years rather than weeks. Moreover, MSCs require modifications to withstand the hostile degenerate‐disc environment; such modifications will require regulatory approval, a long and expensive process; hence modified MSCs may not be readily available for some considerable time and resulting treatments could be very costly.^29^

In addition clinical studies suggesting MSC injections as a possible treatment for LBP, all lack high patient numbers and long‐term results. As not all of them have controls, they are not able to rule out that the positive results reported could arise from a placebo effect. Besides, the level of evidence of all existing studies is low, as there are no results from multicenter, prospective, randomized trials. Ultimately, identification of the right patient for MSC treatment will remain a challenging task.

Hence the treatment of the illustrated case through injection of MSCs cannot thus be recommended. MSC implantation is very unlikely to effect a clinically relevant repair and even if it could, it is unclear whether the patient would benefit from repair of her disc.

### 4.8 Overall conclusion

Among other cell types, MSCs have been tested as a feasible injection therapy for DDD. Despite promising preclinical trials, clinical results are insufficient and multicenter, prospective, randomized trials remain to be conducted. Nevertheless, MSCs hold great regenerative potential due to their proliferation characteristics, their anabolic functionality and inflammation‐modulatory properties. The main challenge for a successful cell‐based IVD therapy can be seen in the harsh microenvironment of the degenerated IVDs that limits the viability and functionality of injected MSCs. However, preconditioning approaches including new emerging techniques in genomics have the potential to prepare MSCs for the survival in IVDs and to take MSCs therapies to the next step.

## CONFLICTS OF INTERESTS

The authors have no conflicts of interest to report.

### Author contributions

The goal of the controversies section is to have renowned experts discuss a defined regenerative treatment option that is close to being available for patients. The ensemble of Markus Loibl, Gianluca Vadala and Karin Wuertz‐Kozak, consisting of two surgeons and one scientist, wrote the first part of the controversy “YES”. Likewise, the ensemble of Jill Urban, Jeremy Fairbank and Siegmund Lang, consisting of one scientist and two surgeons, wrote the second part of the controversy “NO”.
